# Social autopsy study identifies determinants of neonatal mortality in Doume, Nguelemendouka and Abong–Mbang health districts, Eastern Region of Cameroon

**DOI:** 10.7189/jogh.05.010413

**Published:** 2015-06

**Authors:** Alain K. Koffi, Paul–Roger Libite, Seidou Moluh, Romain Wounang, Henry D. Kalter

**Affiliations:** 1 Department of International Health, Johns Hopkins School of Hygiene and Public Health, Baltimore, MD, USA; 2 National Institute of Statistics, Yaoundé, Cameroon; 3 Ministry of Health, Yaoundé, Cameroon

## Abstract

**Background:**

Reducing preventable medical causes of neonatal death for faster progress toward the MGD4 will require Cameroon to adequately address the social factors contributing to these deaths. The objective of this paper is to explore the social, behavioral and health systems determinants of newborn death in Doume, Nguelemendouka and Abong–Mbang health districts, in Eastern Region of Cameroon, from 2007–2010.

**Methods:**

Data come from the 2012 Verbal/Social Autopsy (VASA) study, which aimed to determine the biological causes and social, behavioral and health systems determinants of under–five deaths in Doume, Nguelemendouka and Abong–Mbang health districts in Eastern Region of Cameroon. The analysis of the data was guided by the review of the coverage of key interventions along the continuum of normal maternal and newborn care and by the description of breakdowns in the care provided for severe neonatal illnesses within the Pathway to Survival conceptual framework.

**Results:**

One hundred sixty–four newborn deaths were confirmed from the VASA survey. The majority of the deceased newborns were living in households with poor socio–economic conditions. Most (60–80%) neonates were born to mothers who had one or more pregnancy or labor and delivery complications. Only 23% of the deceased newborns benefited from hygienic cord care after birth. Half received appropriate thermal care and only 6% were breastfed within one hour after birth. Sixty percent of the deaths occurred during the first day of life. Fifty–five percent of the babies were born at home. More than half of the deaths (57%) occurred at home. Of the 64 neonates born at a health facility, about 63% died in the health facility without leaving. Careseeking was delayed for several neonates who became sick after the first week of life and whose illnesses were less serious at the onset until they became more severely ill. Cost, including for transport, health care and other expenses, emerged as main barriers to formal care–seeking both for the mothers and their newborns.

**Conclusions:**

This study presents an opportunity to strengthen maternal and newborn health by increasing the coverage of essential and low cost interventions that could have saved the lives of many newborns in eastern Cameroon.

Progress in reducing global child mortality since 1990 has been encouraging. Yet, reductions in neonatal mortality lag behind survival gains among older children: neonatal deaths accounted for about 44% of 6.3 million under–five deaths in 2013 [1]. There is a global consensus that to achieve the Millennium Development Goal for child survival (MDG–4) to reduce mortality of children under–five years of age by two–thirds between 1990 and 2015, neonatal deaths need to be substantially reduced [2,3].

With an estimated under–five mortality rate in 2012 of 95 deaths per 1000 live births, Cameroon has the 21st highest child mortality rate in the world; neonatal mortality contributed about 30% of these deaths. While neonatal mortality decreased by 20%, from 35 to 28, from 1990 to 2012 [4], the fact that most deaths were from preventable causes suggests that greater attention and investment is required to address neonatal deaths.

In Cameroon, as in many Sub–Saharan African countries, most neonatal deaths occur at home, outside of a medical setting, and are neither registered nor certified as to the cause of death [5]. In addition, data are lacking on the household, community and health system determinants that contribute to these deaths. Making faster progress toward the MGD–4 by reducing preventable causes of deaths will require the country to identify and address these factors contributing to neonatal deaths.

Previous studies have described social factors or determinants that influence neonatal mortality. These factors include poor recognition and understanding of illness signs; socio–cultural traditions regarding maternal and newborn seclusion; lack of access to care due to distance to a facility or provider, lack of transportation means, and limited financial resources for health care or transport; poor quality of care at facilities; and the opportunity costs of missed work or childcare [6–8].

Recently there has been a growing demand for a framework that organizes these factors and a tool to describe them. [9,10]. Two conceptual frameworks, the Pathway to Survival [11] and the Three Delays model [10,12] have been found useful to organize and guide the analysis of these data. In a nutshell, the Pathway to Survival identifies and organizes social, cultural and health system factors that could be modified both inside the home and in the community in order to prevent child illness and return sick children to health [11]. The Three Delays model, which was originally developed to explore barriers to care–seeking for maternal deaths, had been used to understand access to care and care–seeking practices for newborns [12].

“Social autopsy” instruments based on the Pathway to Survival have been developed to collect the data needed to connect the fatal illness or the act of diagnosing or recognizing that illness to a set of socio–demographic, economic, and cultural conditions or factors, thereby making a social “diagnosis” of the deaths [13].

Implicit in the Pathway to Survival is the continuum of care that has been a recurrent theme in the maternal, newborn and child health literature [14]. The interventions reviewed in the continuum of care and related to the Pathway to Survival framework are classified according to service delivery strategies across the continuum and include key preventative interventions, either inside or outside the home, for which a reasonable level of evidence of efficacy is present and that support the child’s wellness [11,15].

The WHO/UNICEF–supported Child Health Epidemiology Reference Group (CHERG) was established in 2001 to provide external technical guidance and global leadership to improve epidemiological estimates of child morbidity and mortality. While most CHERG activities entail gathering and reviewing existing data and building models to develop estimates, verbal/social autopsy (VASA) studies conducted by local partners with CHERG’s technical assistance are collecting new data to directly measure causes of neonatal and child mortality and its determinants in several high priority countries. Thus, a study using an integrated verbal and social autopsy questionnaire was conducted to identify the causes and determinants of neonatal (0–27 days) and young children (1–59 months) deaths in Doume, Nguelemendouka and Abong–Mbang districts, Cameroon from 2007–2010.

The current paper focuses solely on the social autopsy component and aims to shed light on social, cultural, or health systems factors that led to newborn’s death. To the best of our knowledge, there is no prior study of the social autopsy of neonatal deaths in Cameroon. The process of connecting the diagnosis of a fatal illness to a set of social, cultural, or health systems conditions or factors that contributed to the illness has been described previously as the social diagnosis approach [16]. This integrated perspective potentially provides a broader context for social researchers, programs and policy makers to identify modifiable factors that can be addressed or reinforced to improve the design and implementation of maternal, neonatal and child health programs in Cameroon.

## METHODS

### Study sites/districts and sample

The VASA interviews in Cameroon were conducted on deaths identified by a census of 16 954 households undertaken by Population Services International (PSI) from October to December 2010 for the Department of Foreign Affairs, Trade and Development of Canada (DFATD) – funded Home–Based Management of Malaria project in Doume, Nguelemendouka and Abong–Mbang districts. These districts border one another and are located in the East Region of Cameroon. Child mortality in the East region is the second highest in the country at 187 deaths/1000 live births. The survey identified all deaths of children in the prior 10 years from a full birth history of all women age 15–49 years.

To limit issues related to faulty recall, while obtaining an adequate sample size, the VASA study examined deaths of children up to 59 months of age with a 4–year recall period. There were 330 neonatal (0–27 days) deaths and 930 young child (1–59–month old) deaths from 2007 to 2010. Assuming 10% loss due to household relocation and refusals to participate, sample sizes of 330 neonatal deaths and 660 young child deaths (total: 990 deaths) were selected to achieve precision of ±0.05 around the point estimate for the most common cause of young child deaths, and ±0.07 for neonatal deaths, based on an assumed proportion of 50%. Starting with the most recent under–five years old death (whether it was a neonate or child) in all the households and moving back in time, we selected the one most recent under–five years old death (or one at random if there were two or more most recent deaths in the same month) in each household with at least one such death until we had achieved our desired sample sizes in each age group, ie, 330 neonatal deaths and 660 young child deaths. This sampling strategy was previously compared with another one that selected one death at random from each household in the same time period and there were no substantial differences in the child’s age at death or sex or in the respondent’s age. Thus, we retain the approach of selecting the most recent deaths in order to limit the recall period as much as possible, while maintaining the representativeness for each age group within the time period covered by the deaths in that group.

### Data collection tools and VASA interview

The VASA questionnaire blends the Population Health Metrics Research Consortium (PHMRC) verbal autopsy questionnaire to determine the biomedical cause of death, with the CHERG Pathway Analysis social autopsy (SA) questionnaire [17] to inquire about well–child and illness events leading up to a death. The CHERG SA questionnaire updates an earlier Pathway Analysis instrument [18] in order to more fully examine the household, community and health system determinants of neonatal and child mortality, including maternal preventive and curative care in the event of a neonatal death or stillbirth. Unlike the full birth history module used by the PSI census, the VASA allows to further investigate any deaths by asking the respondent if the baby moved, breathed, and cried at birth. When the answer was “No” to all of these items, the software assigned this case to the stillbirth category.

All the illness preventive and curative factors included in the SA questionnaire (**Table 1**) were derived from evidence–based interventions contained in the Lives Saved Tools [19], were judged by a Cochrane review to have good evidence of efficacy, or are among the newborn interventions recommended by the WHO. In addition, where possible, the questions were worded similarly to those in the Demographic and Health Surveys (DHS) [20] in order to allow comparisons of the social autopsy data with similar data for survivors in settings where a recent DHS was conducted.

**Table 1:** CHERG Social autopsy questionnaire content

Family (cultural) factors:
Mother’s age, education, literacy, marital status, age at marriage
Household possessions (VA), husband’s education, breadwinner’s occupation
Pre–pregnancy conditions, ANC provider(s), times & timing of last visit
Knowledge/recognition of and care–seeking for pregnancy, labor and delivery complications
Delivery place, decision maker and factors constraining institutional delivery
Home delivery and newborn care (SBA, delivery surface, cord care, bathing, warmth, breastfeeding)
Infant/child care (smoke exposure, ITN, breastfeeding and nutrition, bottle feeding, pre–illness conditions)
Newborn/infant/child illness recognition, health care–seeking, compliance with treatment & referral advice
Constraints to maternal and child health care–seeking, and constraints to compliance with referral advice for maternal complications and treatment and referral advice for newborn and child illnesses
**Community (social) factors:**
Residence place, duration of continuous residence, and time to reach usual health provider
Social capital (community joint action, helpful persons/groups, denial of services)
**Health systems factors:**
ANC content (BP, urine & blood, counseling on food & care–seeking), TT, ITN, malaria prophylaxis
Delivery care (attendant, partograph use, hygiene, delivery surface)
Newborn care (resuscitation, cord care, bathing, warmth, post–partum counseling, well–baby checks)
Infant/child care (vaccinations, vitamin A)
Quality of maternal and child health care and delivery of services (treatment, referral & reasons for referral for maternal complications and sick children)

CHERG – Child Health Epidemiology Reference Group, VA – Verbal autopsy, ANC – antenatal care, SBA – community-based agents, ITN – insecticide treated nets, BP – blood pressure, TT – tetanus toxoid

The verbal and social autopsy questions are chronologically blended, with social autopsy questions on preventive care asked first, then the verbal autopsy to identify the signs and symptoms of the illness that led to death, followed by social autopsy questions to assess the perception of the illness and care seeking carried out by the caregiver of the deceased person. The VASA questionnaire was developed in English and, for the study in Cameroon, was translated to French, which is understood by the majority of persons in the study area. Local languages, including Mongo–Ewondo and Maka, which are spoken by a large majority but not all local ethnic groups, as well as Baka, Mpoong moon, Onveng and Abakoum also were used. It would be cumbersome to conduct an entire interview in these languages, which are not written. Therefore, only the local terms for key questionnaire items, such as illness signs and symptoms and the names of local traditional and formal health care providers, were translated to the local languages. The first version of this translation was back–translated into French by a separate team of translators. Any discrepancies were reconciled by the translators before the translations of the final list of key terms were validated, phonetically transliterated and inserted into the French questionnaire. Finally, the translations were inserted into a CSPro software application that was developed to enable direct, field–based Computer Aided Personal Interview (CAPI) capture of the VASA interview data on a netbook computer. The CAPI capture allows for automated implementation of skip patterns and internal consistency checks that considerably improve the quality of the interview being conducted.

For the field work, twenty female interviewers who were native speakers of the local languages and had at least a high school education, received 10 days of in–classroom training in the VASA study background, procedures, ethical standards and conduct of the interview on the netbook, followed by 3 days of field practice, all conducted in French and the local languages. The interviewers were split into three groups (one per district) based on their knowledge of the districts, the local languages and their prior involvement during the mortality survey conducted by PSI in 2010. Each team was led by one field supervisor from the National Institute of Statistics of Cameroon and in addition received two field visits by office supervisors during the forty data collection days. The interviewers were trained to select as the respondent the person most knowledgeable of the child’s fatal illness and care provided to the child for the illness. The interview covered the fatal illness from onset to death, including for neonatal deaths, the mother’s pregnancy and delivery. Hence, additional eligible respondents were permitted if necessary. In cases with discordant responses among respondents, the main respondent’s answers outweighed that of the others.

Most of the fieldwork was conducted from 1 April to 15 May, 2012. Review of the collected data revealed 149 cases with large discrepancies between the expected (from the PSI survey) and observed birth dates, ages at death and/or gender of the deceased children. In addition, 71 households were missed (after three attempts of interview) during the first round of data collection for several reasons, such as the family having temporarily or permanently moved outside of the study area or no eligible respondent having been available at the time of the visit. Thus, revisits of these cases were conducted in August 2012 either to ascertain that the VASA interview was conducted for the correct child, to confirm the child’s birth date, age at death and gender, and to re–interview cases as needed, or to attempt to locate the missing family and conduct the interview. Through the revisits, all of the discrepant cases were resolved and only 3 of the 71 first missed interviews were completed with the eligible respondents being present at the time of revisits.

### Data analysis

A descriptive analysis was conducted of the data on preventive and curative care, guided by the coverage of key indicators along the continuum of normal newborn care for well children and the steps of illness recognition and care seeking for child illnesses in the Pathway to Survival model [17–19]. The study added an extended pathway for neonatal illnesses that examined the continuum of normal antenatal care and recognition of and care–seeking for maternal complications during pregnancy, labor and delivery.

Definitions of the maternal complications are found in the **Online Supplementary Document(Online Supplementary Document)**.

For the neonates, in addition to examining the coverage of illness recognition, caregiving, care–seeking and quality health care provision at each step along the Pathway to Survival, we assessed the neonates’ median age in days at illness onset, defined as the age when the first symptoms of the fatal illness were recognized, the median illness duration in days, defined as the time from illness onset till death, and caregivers’ perception of the children’s illness severity at onset, when first deciding to seek formal health care, and at discharge from the first formal provider. Median values are reported for the age at illness onset and the illness duration due to the skewed values for these two variables.

A scoring system was developed to rank caregivers’ perception of their children's illness severity. Perceived severity was ranked 1 (normal/mild), 2 (moderate) or 3 (severe) based on combining the scores for the child’s reported feeding behavior (normally, poorly or not at all), activity level (normal, less active or not moving) and mental status (alert, drowsy or unconscious). For each of these three parameters, a score of 0, 1 or 2.5 was assigned according to the child’s placement along the respective continuum; the individual parameter scores were then combined, with total scores of 0–2, 2.5–3 and 3.5–7.5 being assigned, respectively, to the final ranks of 1, 2 and 3. Feeding behavior, activity level and mental status were used for the scoring system because they reflect children’s actual illness severity as well as mothers’ perception of illness severity and the need to seek health care [21–23]. A balance between possibly discordant biomedical and socio–cultural definitions of certain illness signs was sought by ranking reported unconscious mental status in combination with “feeding normally” or “normally active” as indeterminate (or missing data).

In the development of the severity scoring system, Cronbach’s alpha coefficients [24] were derived to assess the consistency of responses to the score items. The coefficients of the summated scores showed values of 0.84, 0.86 and 0.87 at onset of illness, at time decision to seek care was made, and after leaving the health provider, respectively. Hence, the items in the scores, ie, feeding behavior, activity level and mental status elicited highly consistent responses, justifying the reliability of the summated scores [25].

### Ethical considerations

Ethical clearance for the VASA study was obtained from the Johns Hopkins School of Public Health’s Institutional Review Board and the Cameroon National Research Committee. All respondents provided informed consent before the interview was conducted.

## RESULTS

The VASA interview was completed for 267 (81%) of the 330 neonatal deaths identified by the PSI survey. Of these, 158 were confirmed as neonatal deaths, while 75 and 34 were identified during fieldwork as, respectively, young child deaths and stillbirths. Six of the initially sampled young child deaths were identified as neonatal deaths. As such, the social autopsy analysis was conducted on 164 neonatal deaths.

### Demographic and household characteristics

**Table 2** presents the demographic characteristics of the deceased newborns. Half of the newborns got sick and died within the first 24 hours after birth, and about 71% of the deaths occurred within the first week. Fifty–nine percent of newborn deaths were boys and 41% were girls, for a masculinity ratio of 141. The majority of births (56.1%) and deaths (56.7%) occurred at home. Of the 64 neonates born at a health facility, 40 (63%) died at that facility, ie, they did not leave the facility alive.

**Table 2:** General mortality indicators and demographic characteristics of 164 neonatal deaths, Cameroon, 2007–2010

Characteristics	Frequency (No.)	Percent
Median age at death (in days)	1 (mean 4.0; SD = 5.35)	
Age distribution at death:		
0–6	116	70.7
7–27	47	28.7
Don’t know	1	0.6
Sex:		
Boy	96	58.5
Girl	68	41.5
Masculinity ratio (Boy/Girl ×100)	141	
Place of birth:		
Hospital	52	31.7
Other health provider or facility	12	7.3
On route to a health provider or facility	2	1.2
Home	92	56.1
Other	6	3.7
Delivery mode:		
Vaginal	296	92.6
Caesarian	22	6.9
Don’t know/Missing	2	0.5
Place of death:		
Hospital	50	30.5
Other health provider or facility	5	3.1
On route to a health provider or facility	10	6.1
Home	93	56.7
Other	6	3.6
Born and died at the health facility (without leaving the facility, n = 64)	40	62.5
Median age at illness onset (in days)	1 (mean = 3.0; SD = 4.16)	
Median illness duration (in days)	1 (mean = 2.5; SD = 8.19)	

SD – standard deviation

**Table 3** shows the characteristics of the mother, her domestic partner and the household. Eight in ten (76.2%) mothers of the deceased children were married or were cohabiting with a man at the time of their newborn death. Marriage occurred early for these mothers, with the vast majority (74%) entering into a union before their 20th year. In most of the cases (65%), the breadwinners of the households were farmers or agricultural workers. In this study, only 26% of households had electricity, 21% used in–house piped water supply and 18% used improved sanitation, such as a flush or improved pit toilet. The risk of indoor pollution from cooking fuel was present because 32% of households cooked in the house, 93% used firewood for cooking, and there were on average 6 individuals in the household. It took on average 39 minutes for the caregiver to reach the nearest health center from his/her household. The family had been living in the same community for more than 12 years, yet 48% of mothers did not have anyone to help them during pregnancy or a child illness.

**Table 3:** Characteristics of the mother and her household, 164 neonatal deaths, Cameroon, 2007–2010

Maternal characteristics	Frequency (No.)	Percent
Married or living with a man	125	76.2
Mean age when first married (years):	18.0 (median 17, range 12–33)	
<16	34	27.2
16–19	58	46.4
20+	33	26.4
Mother’s mean age at time of child death (in years):	22.7 (median 21, range 11–45)	
<16	25	15.2
16–19	42	25.6
20–24	39	23.8
25+	53	32.3
Don’t know	5	3.0
Mother’s mean years of maternal schooling:	5.9 (median 6, range: 0–22)	
0–3	20	12.2
4–6	92	56.1
>6	52	31.7
Father’s mean years of schooling:	7.1 (median 6, range: 0–13)	
0–3	4	3.9
4–6	50	48.5
>6	49	47.6
**Household characteristics**		
Main breadwinner:		
Father	114	69.5
Mother	12	7.3
Other	38	23.2
Main breadwinner’s is farmer/agricultural worker	107	65.2
Household size (Mean)	7.0 (median 6, range: 1–24)	
Household has electricity	42	25.6
Use of piped water, –in–house water supply	35	21.3
Use of improved sanitation (flush or improved pit toilet)	30	18.3
Separate room for cooking	112	68.3
Household uses firewood for cooking	152	92.7
Floor of the house made of cement	32	19.5
Mean travel time to usual health facility (min)	41 (median 30, range: 0–270)	
Average time at current residence	12.2 (median 9, range: 0–55)	
**Social capital**		
In last 3 years, community worked together on at least 1 of the following: schools, health, jobs, credit, roads, public transport, water, sanitation, agriculture, justice, security, mosque/church	156	95.1
Mother and her family have never been denied any of the following community	104	63.4
Mother was NOT able to turn to any persons or community groups or organizations for help during the pregnancy or child’s fatal illness	85	51.9

### Maternal and newborn care

**Figure 1:**
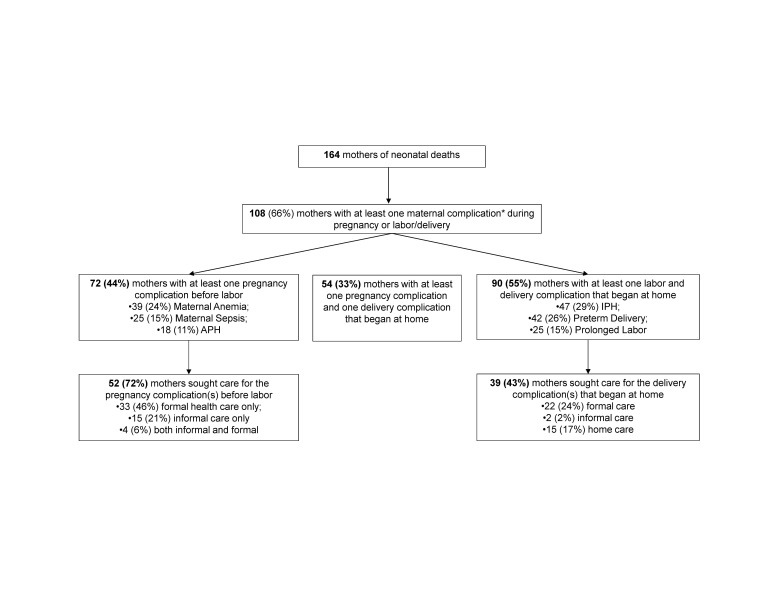
Maternal complications syndromes and care-seeking during the pregnancy and delivery (N = 164). *Maternal complications: Antepartum hemorrhage (APH) – Any vaginal bleeding before labor; Preeclampsia/eclampsia – Puffy face and [blurred vision or severe headache or high blood pressure] and /or Convulsions and no fever and no history of convulsions; Maternal sepsis – Fever and (severe abdominal pain or smelly vaginal discharge or foul smelling liquor); Maternal anemia – (Severe anemia or pallor and shortness of breath) and (too weak to get out of bed or fast or difficult breathing); Intrapartum hemorrhage (IPH) – Excessive bleeding during labor or delivery; Preterm delivery – Less than 9 months; Prolonged labor – Labor for 12 hours or more

**Figure 1** presents maternal complications and care–seeking during the pregnancy and/or delivery. During their pregnancy (before labor), 24%, 15% and 11% of the mothers suffered from anemia, sepsis and antepartum hemorrhage, respectively. The main labor/delivery complications that started at home comprised intra–partum hemorrhage (29%), preterm delivery (26%), and prolonged labor (15%). Overall, just half of the mothers (37 out of 72 or 52%) with a pregnancy complication sought some formal care. Less than a quarter (24%) of the 90 mothers with at least one labor and delivery complication that began at home sought some formal care.

Among the 64 neonates born at a health facility, 50% of the mothers of 40 newborns who did not leave alive had at least one labor/delivery complication that started at home, compared to 46% (with at least one labor and delivery complication that started at home) of the 24 who left alive, and the difference was not statistically significant. Among the 40 mothers whom babies were born and died a health facility (without leaving alive) 12 (30%) suffered from anemia during pregnancy; and during labor or delivery, 17 (42.5%) and 11 (27.5%), reported on intra–partum hemorrhage and preterm–delivery complications, respectively.

**Figure 2:**
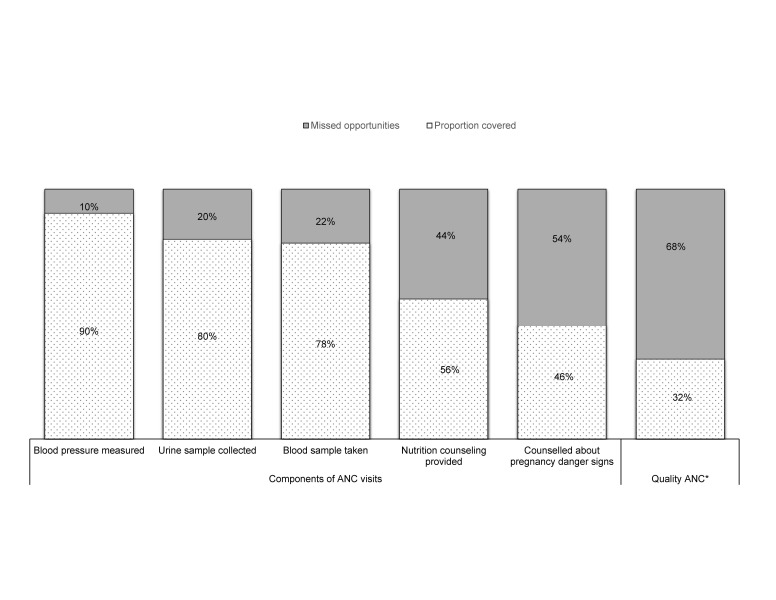
Quality gap for at least one antenatal care visit (N = 125). For women who went to at least one antenatal care (ANC) visit (N = 125), a quality gap (or missed opportunity) exists and represents the difference between the expected maximum coverage and the actual coverage proportion. *Quality ANC includes blood pressure checked, urine and blood tested, nutrition counsels, and counsels about danger signs.

**Figure 2** shows the components of the antenatal care among mothers who completed at least one visit. During the pregnancy of the deceased neonates, 39 (24%) of the mothers did not benefit from any antenatal care (ANC). For women who went to at least one ANC visit, a quality gap exists because some of them did not receive all of the ANC components, including blood pressure measurement, urine and blood sample tests, and counseling on proper nutrition and pregnancy danger signs. Thus, ANC of “quality” suggests all of the ANC components were provided, ie, blood pressure checked, urine and blood tested, nutrition counsels, and counsels about danger signs. Hence, of the 125 mothers who went to a health provider for at least one ANC visit, only 32% of the mothers received ANC of “quality”. And the quality gap or missed opportunity ranged from 10% for blood pressure checked to 54% for counselling about danger signs.

**Figure 3:**
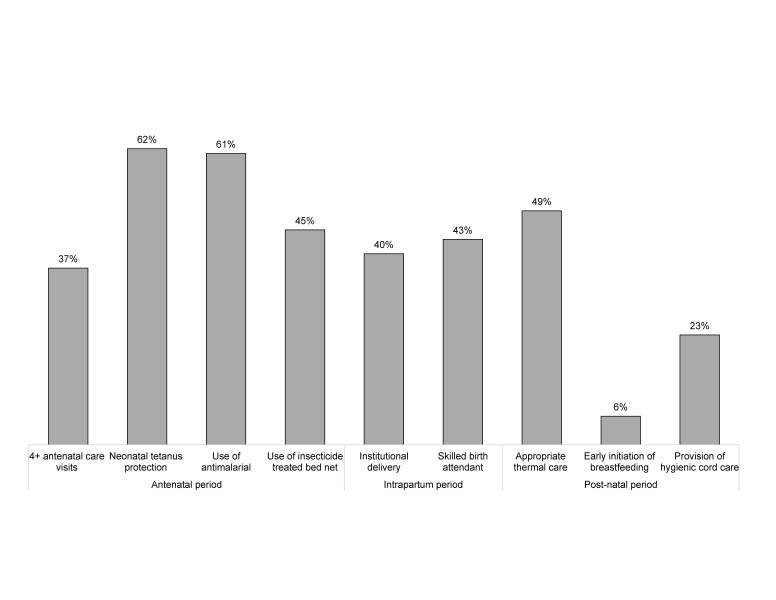
Preventive care of the mothers and newborns (N = 164).

**Figure 3** shows the preventive care received by mothers and newborns along the continuum of care. Just 37% of the mothers benefited from the recommended four or more ANC visits. Forty percent of mothers of deceased neonates delivered at a health facility. Overall, 43% of the 164 mothers were assisted by skilled birth attendants, ie, doctors, nurses or midwives. Among those who survived the first day of life, about 49% received appropriate thermal care consisting of immediate warming, drying and wiping, wrapping in a blanket, skin to skin contact with the mother or being placed in an incubator, plus bathing delayed for more than 24 hours after birth. Overall, 33.5% of the mothers breastfed their newborns in the first 24 hours after birth (result not shown). Yet, early initiation of breastfeeding, ie, within one hour after birth, was lower at 6%. And 23% were provided hygienic cord care. Hygienic cord care suggests a new boiled razor blade from the delivery kit was used for cutting the cord, a clean boiled piece of thread from the delivery kit was used for tying the cord and nothing was applied to the umbilical cord stump after birth or in case something was applied, either alcohol or other antiseptic or antibiotic ointment in cream or powder form was applied.

**Figure 4:**
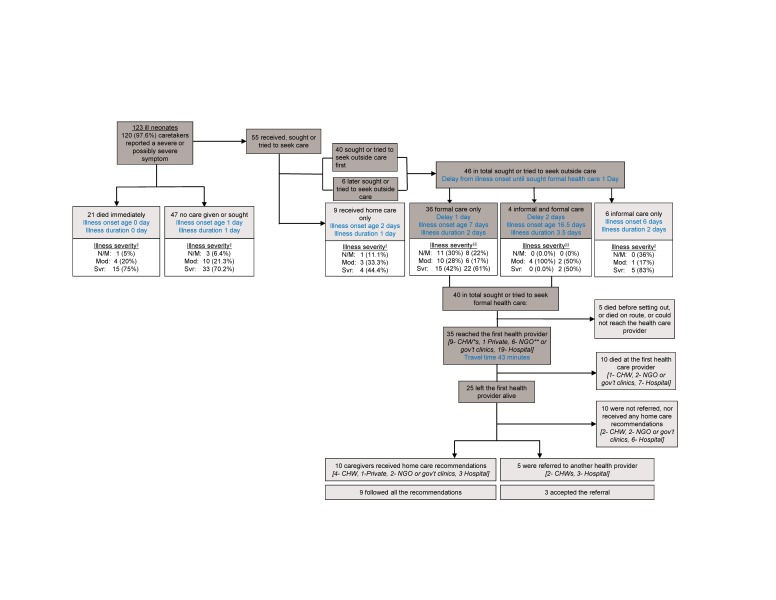
The “Pathway to Survival” for 123 neonatal deaths (born at home or left the delivery facility alive). ^§^Illness severity at onset; ^§§^Illness severity at onset and when caregiver decided to seek formal care; N/M – normal/mild, Mod – moderate, Svr – severe; *CHW – Community Health Worker, **NGO – Non-governmental organization.

**Figure 4** shows the study findings based on the Pathway to Survival model for 123 newborns with an opportunity for careseeking, including those who were born at home or were born in a health facility and left the facility alive. Forty (24%) of the 164 neonates included in the study sample died at the facility where they were delivered, and one was born at home but was missing all information of careseeking.

At the onset of the 123 newborns’ fatal illnesses, 98% of caregivers could recognize and report a severe or possibly severe symptom. Yet, only 55 (44.7%) caregivers provided home care or sought or tried to seek outside care for their newborn child. Twenty–one (17.1%) newborns “died immediately” and no care was given or sought for another 47 (38.2%). Most (70–75%) of the neonates in these last two groups were ranked as being severely ill at the onset of their illness, compared to 44% of those for whom any care was given or sought. Understandably, in the group of those who died “immediately”, the age at illness onset and duration were both 0–day, meaning the newborns were born and died quickly (or “immediately”) the same day of birth. Conversely, for newborns in the group that received no care, the illness occurred on the second day of life and lasted just 1 day.

Of the 55 neonates who received, sought or tried to seek care, 40 first sought care outside the home, 15 first received care inside the home, and 6 of these 15 later sought or tried to seek outside care. Among the 46 who sought any outside care, 36 sought formal care only, 4 sought both informal and formal care, and 6 sought informal care only. The median delay from the onset of illness until formal health care seeking was 1 day. The delay to seeking formal care when both informal and formal care were sought (2 days) was greater (*P* < 0.05) than the delay when formal care only was sought (1 day).

Of the 40 newborns for whom formal care was sought, more than half were already severely ill at the time the caregiver decided to seek care. Five neonates did not reach the health facility because they died either before setting out or on route or could not reach the health provider. The remaining 35 newborns reached the first health provider after on average 43 minutes travel time. Nine went to a community health worker (CHW), 1 to a private doctor or clinic, 6 to a non-governmental- organization (NGO) or government clinic, and 19 to an NGO or government hospital.

More than one–third (37%, n = 7) that went to an NGO or government hospital died without leaving that hospital. Another child died at the CHW and 2 others at an NGO or government clinic. Of the 25 who left the provider alive, 10 received home care recommendations, 5 were referred to another health care provider, and 10 were sent home without being referred or given any home care recommendations.

It is worth mentioning that of the 8 newborns who went and left a CHW alive, just 2 were referred to a second provider. Yet, 4 of the 6 caregivers who were not referred reported that their newborns were severely ill at discharge from the CHWs.

**Figure 4** also shows that when recommendations were received, most caregivers (9 out of 10) followed them all. Similarly, when the 5 neonates were referred, 3 of them accepted the referral and went to a second health provider.

**Figure 5:**
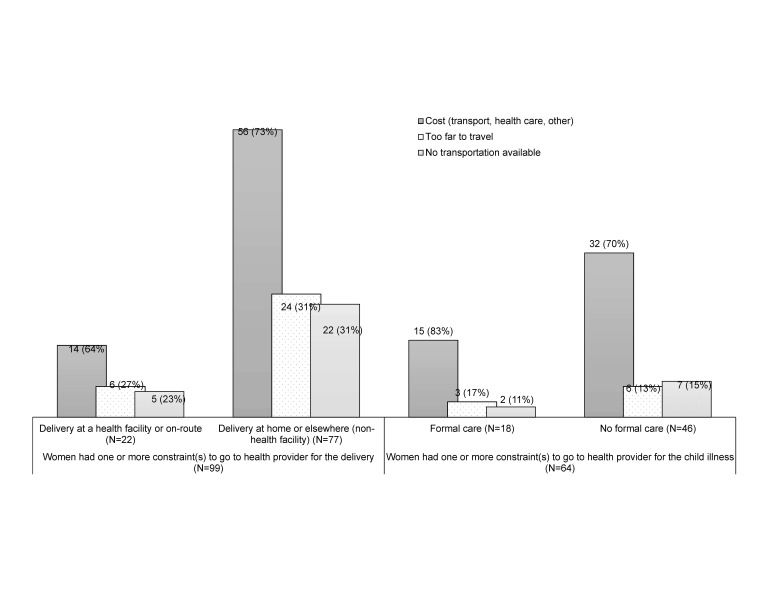
Main care–seeking constraints for the delivery and for the neonatal illness.

**Figure 5** explores the care–seeking constraints for the delivery and for the neonatal illness. Of the 99 mothers that reported concerns or problems for delivering at a health facility, the majority (77.8%, n = 77) delivered at home or another place other than a health facility. Similarly, of the 64 caregivers that mentioned one or more constraints for seeking formal care for their newborn’s illness, the majority (71.9%, n = 46) sought informal care only or did not seek any care at all. Among the problems reported both during delivery and during the newborn fatal illness, the cost for transport and/or health care, distance to reach the provider and lack of transportation emerged as the most important constraints.

## DISCUSSION

The objective of this study was to explore the household, community and health system determinants of neonatal mortality in Doume, Nguelemendouka and Abong–Mbang health districts, in Eastern Region of Cameroon from 2007–2010.

### Demographic and household characteristics

Our study findings showed that the vast majority of the deceased newborns lived in poor households lacking basic commodities such as electricity, improved sanitation and clean water. The households were crowded and more than nine–tenths used firewood for cooking. Most families’ breadwinners were farmers. These impoverished conditions of households in this study could have contributed to the neonatal deaths. Indeed, previous studies have highlighted an increased risk of neonatal deaths of poor families because they face more challenges in accessing timely, high quality health care compared to wealthier families [26,27].

Differences in mortality rates for male and female children are highest during the neonatal period. Baby girls have a lower mortality rate than boys in societies where equal care is offered to both sexes [28].Similarly in the three study districts, the number of newborn deaths was 1.4 times higher among male than female children.

The majority of the deceased newborns were born to young mothers less than 20 years of age. Elsewhere, complications of pregnancy and childbirth have been described as the leading causes of perinatal death of babies born to mothers under 20 years of age, as adolescent mothers’ babies are more likely to be of low birth weight and/or to be born prematurely [29]. Understandably, WHO has developed extensive guidelines to prevent early pregnancy and its poor health outcomes by preventing marriage of adolescents, by increasing knowledge and understanding of the importance of pregnancy prevention, by increasing the use of contraception and by preventing coerced sex [30].

### Maternal complications and care during pregnancy, labor and delivery, and immediate post–neonatal care

Huda et al. estimated that complications during labor and delivery increased the risk of perinatal mortality deaths [31]. Globally, complications from preterm births, intrapartum–related disorders or birth asphyxia, and infections have been consistently reported as the leading causes of neonatal deaths [32,33]. In the current study, labor and delivery complications that started at home occurred in more than half of the pregnant mothers of the deceased newborn. Yet, very few sought formal care when those complications occurred, thereby adding to the increased risk of deaths of their newborns. Therefore, early recognition, immediate care–seeking and referral (when necessary) of women with obstetric complications should be a program priority. Equally, basic and comprehensive emergency obstetric care that includes essential and emergency newborn care and newborn resuscitation is marked by a series of core competencies defined by the World Health Organization (WHO), the International Council of Midwives (ICM), and the International Federation of Gynecology and Obstetrics (FIGO) [34].

This study also revealed that one in five mothers of the deceased newborns did not receive any antenatal care, thereby, significantly increasing the risk of perinatal mortality [29]. ANC is a health service package that links the woman and her family to the health system during pregnancy. Not only does it directly improve the survival and health of babies by reducing stillbirths and neonatal deaths, but it also constitutes an entry point for health facilities contacts with women [35].

In addition, while coverage of at least one antenatal care visit, at 79%, was relatively high in these three districts in Cameroon, coverage of the recommended minimum four–visits, at 37%, was much lower. And even when they had contact with the health system during their pregnancy, more than two–thirds of mothers did not receive high quality antenatal care. Indeed, more than one antenatal visit is required for a health provider to record a mother’s medical history, assess her individual needs, conduct screening tests, provide advice and guidance on pregnancy and delivery educate on self–care during pregnancy, identify conditions detrimental to health during pregnancy, provide first–line management, and referral if necessary [36,37]. This quality gap found in our study demonstrates key missed opportunities that could have saved several lives within the health system.

The VASA study also highlighted the low coverage of other key interventions along the continuum of care, such as delivery at health facility, skilled birth attendants, hygienic cord care, appropriate thermal care and early initiation of exclusive breastfeeding. Previously, health facility delivery has been found to reduce the risk of neonatal mortality by 29% in developing countries [38]. And if practiced routinely at both community and facility levels, it is known that hygienic cord care, proper thermal care and early initiation of breastfeeding practices, could reduce newborn deaths by up to 30% [39].

### Newborn care during fatal illness

At the time caregivers first noticed the illness, 65% of neonates were severely ill, meaning they could not eat at all, they were unconscious/or and they could not move. Timing is critical to providing neonates with appropriate care at the onset of illness, and delays in deciding to seek care can have significant consequences [33]. While the first, fundamental, step in taking this decision is for caregivers to be able to recognize illness danger signs, this can be particularly challenging in the neonate due to the lack of specific symptoms [40–42]. In addition, careseeking was delayed for several neonates who became sick after the first week of life and whose illnesses were less serious at the onset until they became more severely ill. Other studies have described interventions to promote maternal recognition of neonatal illnesses and careseeking before the child becomes severely ill [43,44].

In Cameroon, the community–based Integrated Management of Childhood Illness (C–IMCI) strategy was also designed to focus on the major causes of death in children under–five through improving case management skills of health workers, strengthening the health system, and addressing family and community practices. However, C–IMCI modules, like in other developing countries, did not originally include care of the sick newborn. Hence, the fact that two–thirds of the newborns that went to a CHW were severely ill, yet left without being referred, echoes the need to scale up newborn health interventions using the IMCI strategy. Indeed, current international opinion suggests that incorporating newborn algorithms in IMCI and strengthening the components of the strategy related to the health system and community will directly impact newborn health [45].

### Barriers to maternal and newborn care

Barriers to care extend beyond the health service and include issues such as distance, beliefs, financial and transport constraints. The findings show that unaffordable costs for transportation and health care were key barriers to seeking health care, both for pregnancy and labor/delivery complications and for newborn fatal illnesses. These findings suggest a need to mitigate the costs of care–seeking and lack of means of transport. One possibility is to provide finance, either at the central or at the local level, to cover the costs of transport, and user fees. Conditional cash transfers programs, community insurance schemes, coupons and vouchers, and facility funds for cost reimbursements are possible mechanisms [46–48]. One alternative is to provide subsidized transport services to get newborns to hospital, and another, on a more ambitious scale, is the building, or repairing, of local roads and bridges to help people get to the health facilities.

### Study limitations

This study had some limitations. Given the recall period of about 4 years, added to the fact that the respondents were the main caregivers of the deceased newborns, it is possible that the data may have been affected by different types of biases, including recall bias of past events and the likelihood of providing socially desirable answers to sensitive questions. However, the conversational and prompting modes used during the face–to–face interviews may have led to better overall recall of events. In addition, the study was conducted in a small area of the Eastern region of Cameroon, rendering it difficult to generalize the findings to the entire country. Given the diversity of cultures and population in Cameroon, a national study could offer a clearer picture of the entire country. Last, the inclusion of a control group would have allowed the analysis to test whether or not there were significant differences between the coverage of interventions among cases (deceased newborns) and controls (alive newborns). However, the lack of a comparison group in social autopsy studies is common and not so necessary since we are studying proven interventions that should be accessible to all pregnant mothers and newborns.

## CONCLUSION

The social autopsy study provided a unique opportunity to review the coverage of essential interventions for deceased newborns and their mothers along the continuum of care, to identify the breakdowns within the Pathway to Survival that led to the newborn deaths and to examine the care–seeking barriers during delivery and the newborns’ fatal illnesses that contributed to the deaths. Newborns are vulnerable and dependent upon their families for survival, but poor families, especially those in rural, peri–urban and remote areas, such as in Doume, Nguelemendouka and Abong–Mbang health districts, in Eastern Region of Cameroon, do not have the resources necessary to care for their newborns.

Maternal health and well–being play an important role in newborn survival, pointing to the need to strengthen the continuum of care for maternal, newborn and child health. While most women access the formal health system during pregnancy, the number of visits and quality of care must be increased in order to address the gap between service utilization and need around the time of childbirth and the early postnatal period, during which most newborn deaths occur. Challenges at the facility level must be addressed. Yet, the fact that the majority of births and newborn deaths happen at home in these districts means that successful community partnerships, social mobilization, and health education and behavior change communication is also required to improve knowledge of pregnancy related and newborn or child illness symptoms and careseeking behaviors in order to save lives.
