# Supplementary material for: Social autopsy study identifies determinants of neonatal mortality in Doume, Nguelemendouka and Abong–Mbang health districts, Eastern Region of Cameroon
Source: J Glob Health. 2015 May 19;5(1):010413. doi: 10.7189/jogh.05.010413 (PMC4459092; doi:10.7189/jogh.05.010413)
Supplement: Online Supplementary Document [file jogh-05-010413-s001.pdf]

Online Supplementary Document

Koffi et al. Social autopsy study identifies determinants of neonatal mortality in Doume, Nguelemendouka and Abong–Mbang health districts, Eastern Region of Cameroon

J Glob Health 2015;5:010413

DEFINITIONS OF MATERNAL COMPLICATIONS

The pregnancy complications include:

Antepartum hemorrhage: Any vaginal bleeding before labor;

Preeclampsia/eclampsia: Puffy face and [blurred vision or severe headache or high blood pressure] and /or Convulsions and no fever and no history of convulsions;

Maternal sepsis: Fever and (severe abdominal pain or smelly vaginal discharge);

Maternal anemia: (Severe anemia or pallor and shortness of breath) and (too weak to get out of bed or fast or difficult breathing);

Gestational Diabetes: Diabetes that started during pregnancy before labor began;

Premature rupture of the membranes: Water broke 6 hours or more before labor began;

Malaria: Convulsions and fever

The labor and delivery complications include the following:

Intrapartum hemorrhage: Excessive bleeding during labor or delivery;

Preeclampsia/eclampsia: same as for pregnancy;

Maternal anemia: same as for pregnancy;

Maternal sepsis: Fever and (severe abdominal pain or smelly vaginal discharge or foul smelling liquor);

Preterm delivery: Less than 9 months;

Prolonged labor: Labor for 12 hours or more;

Malaria: same as for pregnancy.
